# Supplementary material for: Silencing the Mitochondrial Gatekeeper VDAC1 as a Potential Treatment for Bladder Cancer
Source: Cells. 2024 Apr 4;13(7):627. doi: 10.3390/cells13070627 (PMC11012128; doi:10.3390/cells13070627)
Supplement: Supplementary file 1 [file cells-13-00627-s001.zip › cells-2900245-supplementary.pdf]

Supplementary Materials

Table S1. Antibodies used in this study

Antibodies against the indicated protein, their catalogue number, source, and the dilutions used in immunohistochemistry (IHC), immunoblot (WB), and immunofluorescence (IF) staining are presented.

Antibody	Source and Cat. No.	Dilution		
		IHC	WB	IF
Rabbit monoclonal anti-VDAC1	Abcam, Cambridge, UK, ab15895	1:400	1:5000	1:1000
Mouse monoclonal anti-VDAC1	Abcam, Cambridge, UK Ab186321	1:500	1:5000	1:1000
Rabbit monoclonal anti-KI-67	Abcam, Cambridge, UK ab16667	1:100		1:100
Mouse monoclonal anti-β-actin	Millipore, Billerica, MA, MAB1501		1:20000	
Rabbit polyclonal anti-citrate synthase (CS)	Abcam, Cambridge, UK ab96600		1:5000	1:250
Rabbit monoclonal anti-Cyclin D1	Abcam, Cambridge, UK, ab134175		1:5000	
Rabbit polyclonal anti-COX IV	Abcam, Cambridge, UK ab16056			1:500
Rabbit monoclonal anti-GLUT1	Abcam, Cambridge, UK, ab115730			1:200
Mouse monoclonal anti-GAPDH	Abcam, Cambridge, UK, ab9484			1:200
Rabbit polyclonal anti-CD31	Abcam, Cambridge, UK, ab28364		1:2000	
Rabbit polyclonal anti-CD-68	Abcam, Cambridge, UK, ab125212			1:2000
Rabbit polyclonal anti-VEGF-B	Santa Cruz, TX, USA, sc-80442			1:200
Mouse monoclonal anti-ATP5a	Abcam, Cambridge, UK, ab14748		1:1000	1:300
Rabbit monoclonal anti-HK-I	Abcam, Cambridge, UK, ab150423			1:200
Rabbit monoclonal anti-LDH	Abcam, Cambridge, UK, ab52488			1:400
Mouse monoclonal anti-Vimentin	Abcam, Cambridge, UK, Ab8978			1:200
Rabbit monoclonal anti-E-Cadherin	Cell signaling, Danvers, MS, USA 3195S			1:200
Mouse monoclonal anti-N-Cadherin	Santa Cruz, TX, USA, sc-393933			1:200
Rabbit monoclonal anti-Cytokeratin14	Abcam, Cambridge, UK Ab181595	1:500		
Rabbit monoclonal anti-ALDH1A1	Abcam, Cambridge, UK Ab52492	1:150	1:2000	
Rabbit polyclonal anti-α-SMA	Abcam, Cambridge, UK Ab5694		1:2000	1:200
Rabbit monoclonal anti-CD44	Abcam, Cambridge, UK, ab243894			1:300
Rabbit polyclonal Anti-SOX2	Abcam, Cambridge, UK, ab97959			1:500
Anti-mouse IgG, Alexa Fluor 488	Abcam, Cambridge, UK, ab150113			1:1000
Anti-rabbit IgG, Alexa Fluor 555	Abcam, Cambridge, UK, ab150078			1:1000
Donkey anti-mouse HRP	Abcam, Cambridge, UK, ab98799		1:10000	
Goat anti-rabbit HRP	Promega, Wisconsin, W4018		1:10000	

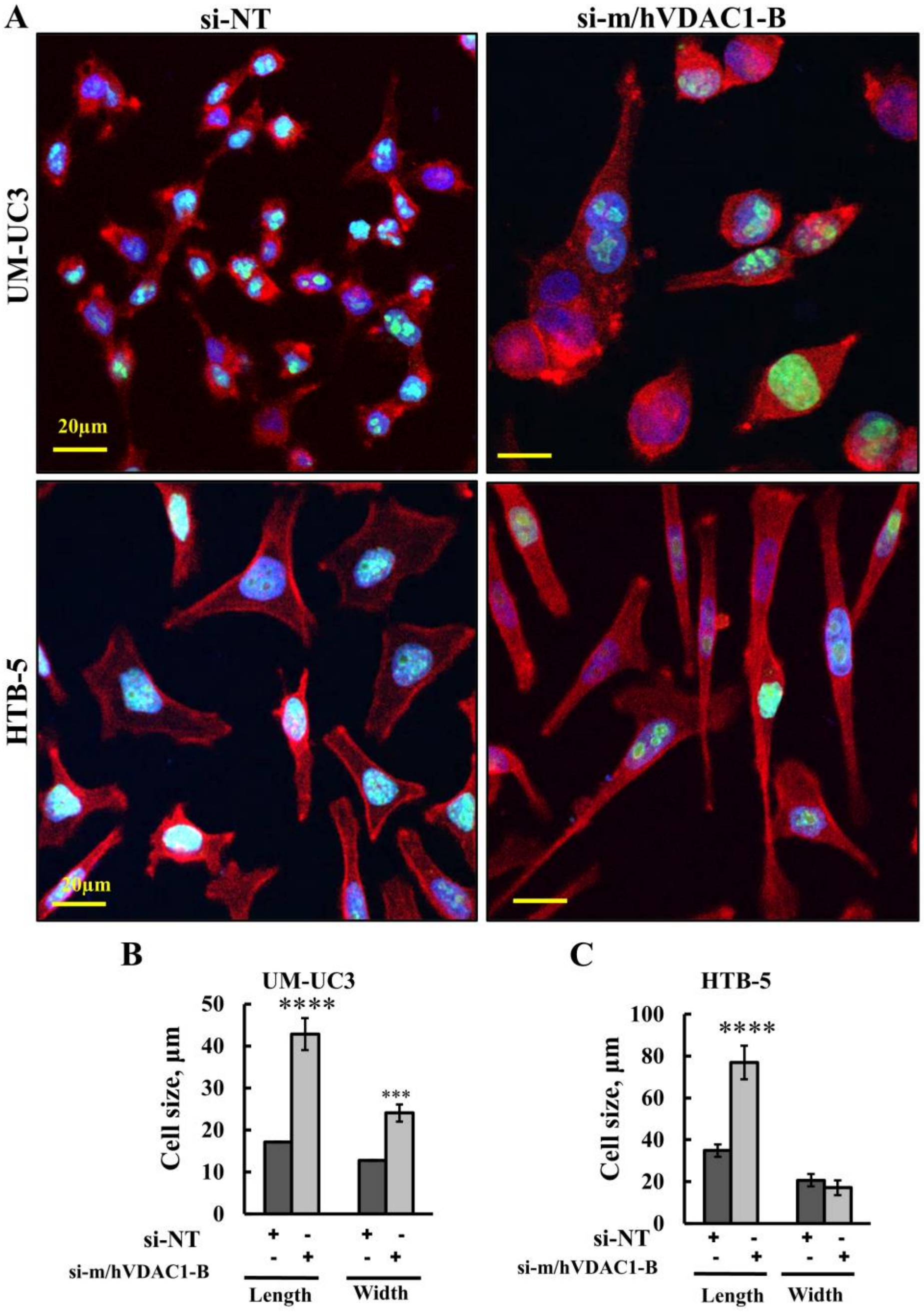

Figure S1. Silencing VDAC1 expression impact cell morphology.

Cells were treated with 50nM si-m/h-VDAC1 or si-NT, and 72h post-transfection were stained for F-actin using phalloidin or for Ki-67 using specific antibodies and visualized using a confocal microscope (**A**). Cell size, length and width were measured (n=30) and presented for both UN-UC3 and HTB-5 cell lines treated with si-NT or with si-m/h-VDAC1-B (**B,C**). Results are the means ± SEM, ***$p < 0.001$, ****$p < 0.0001$.

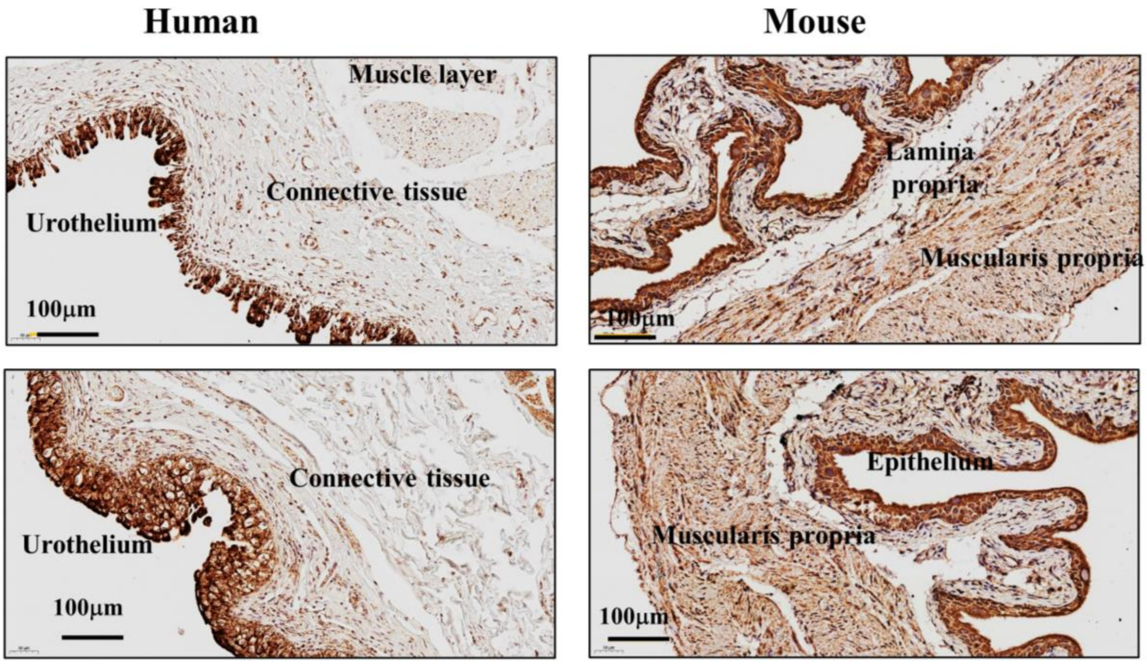

Figure S2. VDAC1 in healthy human and mouse bladder epithelium.
IHC staining of VDAC1 in sections of bladder from two healthy humans or mice, showing its high expression in the epithelium of these sections.
